# Assessment of glycan interactions of clinical and avian isolates of *Campylobacter jejuni*

**DOI:** 10.1186/1471-2180-13-228

**Published:** 2013-10-14

**Authors:** Christopher J Day, Greg Tram, Lauren E Hartley-Tassell, Joe Tiralongo, Victoria Korolik

**Affiliations:** 1 Institute for Glycomics, G26, Griffith University Gold Coast Campus, Queensland 4222, Australia

**Keywords:** Host-bacterial interactions, Glycan array, Glycoconjugates

## Abstract

**Background:**

*Campylobacter jejuni* strain 11168 was demonstrated to have a broad specificity for eukaryotic surface glycosylation using glycan array analysis. The initial screen indicated that sialic acid and mannose are important binding partners after environmental stress, while galactose and fucose structures are likely to be involved in persistent infection.

**Results:**

In this broader study, five additional human/clinical isolates and six chicken isolates were fully assessed to determine their glycan binding capacity using an extended glycan array. *C. jejuni* 11168 was rescreened here due to the presence of glycoaminoglycan (GAG) and other structures that were not available on our previous glycan array. The current array analysis of additional *C. jejuni* strains confirmed the growth condition dependent differences in glycan binding that was previously observed for *C. jejuni* 11168. We noted strain to strain variations, particularly for the human isolates *C. jejuni* 520 and 81116 and the chicken isolate *C. jejuni* 331, with the majority of differences observed in galactose, mannose and GAG binding. Chicken isolates were found to bind to a broader range of glycans compared to the human isolates, recognising branched mannose and carageenan (red seaweed) glycans. Glycan array data was confirmed using cell-based lectin inhibition assays with the fucose (UEA-I) and mannose (ConA) binding lectins.

**Conclusions:**

This study confirms that all *C. jejuni* strains tested bind to a broad range of glycans, with the majority of strains (all except 81116) altering recognition of sialic acid and mannose after environmental stress. Galactose and fucose structures were bound best by all strains when *C. jejuni* was grown under host like conditions confirming the likelihood of these structures being involved in persistent infection.

## Background

Glycan or carbohydrate based host-bacterial interactions are crucial for the initiation of both disease and colonisation of many bacteria species [1-4]. Specifically, the ability to recognise a broad range of host cell surface glycosylation has been shown to be crucial for the adherence and infectivity of *C. jejuni*[3,4]. *In vivo,* fucosylated glycans present on human breast milk proteins and free fucosylated oligosaccharides can reduce the incidence of *C. jejuni* infections in breastfeeding infants [5,6]. While *in vitro*, blocking the surface glycans with lectins to fucosylated and terminal galactose structures can completely inhibit the adherence of *C. jejuni* to Caco-2 cells [3].

Glycan array analysis of *C. jejuni* 11168 found that binding of *C. jejuni* to mannosylated and sialylated glycoconjugates was dependent on the growth or maintenance conditions of the bacteria [3]. After exposure of *C. jejuni* to environmental stress (normal oxygen and room temperature) the bacteria were found to bind extensively to mannosylated and sialylated glycoconjugates. This binding was eliminated when the bacteria were grown under microaerobic conditions at either 37°C or 42°C; at these conditions binding to galactose and fucose predominated [3].

Within the Epsilon proteobacteria a complete spectrum of glycans involved in host bacterial interactions has been determined for *Helicobacter pylori*. Like *C. jejuni, H. pylori* exhibits broad complexity in carbohydrate-binding specificities. It has been proposed for *H. pylori* that initial interactions with host tissues may be achieved through binding to the normal gastric epithelium which expresses non-sialylated glycoconjugates such as the Lewis B antigen through the action of the lectin BabA [2,7,8]. In addition, persistence of *H. pylori* infection appears to be mediated through the binding of the lectin SabA to the sialylated diseased epithelium of the chronically infected stomach [2,8,9]. In contrast, the initial interactions for *C. jejuni* 11168, appear to be to highly sialylated and mannosylated structures such as those found on human glycoprotein MUC1, abundant in human intestinal mucosa [3,4,8,10]. While persistent *C. jejuni* infection in crypts of the intestinal epithelium seems to depend on fucose and galactose, structures more readily found on the gel forming mucins such as MUC2 [3,4,8].

*C. jejuni* has a broad host range, infecting a wide range of both avian and mammalian hosts. Within eukaryotes there are numerous differences between the types of surface glycans present with differences in sialic acids (Neu5Ac/Neu5Gc) expressed, level and linkages type to fucose and the degree of terminal α/β linkages to terminal galactose residues [11-14]. *C. jejuni* can be either infectious or commensal in different hosts, with disease typically observed in mammals and commensal relationships with avian species. Whether or not the host temperature or glycan expression may play a role in this is still to be elucidated. In this study we analysed the glycan binding profile of twelve strains of *C. jejuni* isolated from human and avian hosts with differing invasive profiles to determine if there are any glycan binding differences between invasive and non-invasive *C. jejuni*.

## Results

### Glycan array analysis of *C. jejuni* strains

Glycan array screening was performed on *C. jejuni* strains grown under different conditions to determine the glycan binding specificity for each strain tested. Each of the twelve *C. jejuni* strains was found to recognise a large variety of glycoconjugates present on the array (Tables 1, 2, 3 and 4; see Additional file 1: Table S1 for full list and structures of glycans).

**Table 1:** **Terminal galactose binding from the glycan array analysis of twelve *C. jejuni *strains**

**Glycan ID**	**Human**																		**Chicken**																	
	**11168**			**351**			**375**			**520**			**81116**			**81–176**			**331**			**008**			**019**			**108**			**434**			**506**		
	**RT**	**37**	**4**	**RT**	**37**	**42**	**RT**	**37**	**42**	**RT**	**37**	**42**	**RT**	**37**	**42**	**RT**	**37**	**42**	**RT**	**37**	**42**	**RT**	**37**	**42**	**RT**	**37**	**42**	**RT**	**37**	**42**	**RT**	**37**	**42**	**RT**	**37**	**42**
1A	**+**	**+**	**+**	**+**	**+**	**+**	**+**	**+**	**+**	**+**	**+**	**+**	**+**	**+**	**+**	**+**	**+**	**+**	**+**	**+**	**+**	**+**	**+**	**+**	**+**	**+**	**+**	**+**	**+**	**+**	**+**	**+**	**+**	**+**	**+**	**+**
1B	**+**	**+**	**+**	**+**	**+**	**+**	**+**	**+**	**+**	**+**	**+**	**+**	**+**	**+**	**+**	**+**	**+**	**+**	**-**	**-**	**-**	**+**	**+**	**+**	**+**	**+**	**+**	**+**	**+**	**+**	**+**	**+**	**+**	**+**	**+**	**+**
1C	**+**	**+**	**+**	**+**	**+**	**+**	**+**	**+**	**+**	**+**	**+**	**+**	**+**	**+**	**+**	**+**	**+**	**+**	**-**	**-**	**-**	**+**	**+**	**+**	**+**	**+**	**+**	**+**	**+**	**+**	**+**	**+**	**+**	**+**	**+**	**+**
1D	**+**	**+**	**+**	**+**	**+**	**+**	**+**	**+**	**+**	**+**	**+**	**+**	**+**	**+**	**+**	**+**	**+**	**+**	**+**	**+**	**+**	**+**	**+**	**+**	**+**	**+**	**+**	**+**	**+**	**+**	**+**	**+**	**+**	**+**	**+**	**+**
1E	**+**	**+**	**+**	**+**	**+**	**+**	**+**	**+**	**+**	**+**	**+**	**+**	**+**	**+**	**+**	**+**	**+**	**+**	**+**	**+**	**+**	**+**	**+**	**+**	**+**	**+**	**+**	**+**	**+**	**+**	**+**	**+**	**+**	**+**	**+**	**+**
1 F	**+**	**+**	**+**	**+**	**+**	**+**	**+**	**+**	**+**	**-**	**-**	**-**	**+**	**+**	**+**	**+**	**+**	**+**	**+**	**+**	**+**	**+**	**+**	**+**	**+**	**+**	**+**	**+**	**+**	**+**	**+**	**+**	**+**	**+**	**+**	**+**
1G	**+**	**+**	**+**	**+**	**+**	**+**	**+**	**+**	**+**	**+**	**+**	**+**	**+**	**+**	**+**	**+**	**+**	**+**	**+**	**+**	**+**	**+**	**+**	**+**	**+**	**+**	**+**	**+**	**+**	**+**	**+**	**+**	**+**	**+**	**+**	**+**
1H	**+**	**+**	**+**	**+**	**+**	**+**	**+**	**+**	**+**	**+**	**+**	**+**	**+**	**+**	**+**	**+**	**+**	**+**	**+**	**+**	**+**	**+**	**+**	**+**	**+**	**+**	**+**	**+**	**+**	**+**	**+**	**+**	**+**	**+**	**+**	**+**
1I	**+**	**+**	**+**	**+**	**+**	**+**	**+**	**+**	**+**	**+**	**+**	**+**	**+**	**+**	**+**	**+**	**+**	**+**	**-**	**-**	**-**	**+**	**+**	**+**	**+**	**+**	**+**	**+**	**+**	**+**	**+**	**+**	**+**	**+**	**+**	**+**
1 J	**+**	**+**	**+**	**+**	**+**	**+**	**+**	**+**	**+**	**+**	**+**	**+**	**+**	**+**	**+**	**+**	**+**	**+**	**-**	**-**	**-**	**+**	**+**	**+**	**+**	**+**	**+**	**+**	**+**	**+**	**+**	**+**	**+**	**+**	**+**	**+**
1 K	**+**	**+**	**+**	**+**	**+**	**+**	**+**	**+**	**+**	**-**	**-**	**-**	**+**	**+**	**+**	**+**	**+**	**+**	**-**	**-**	**-**	**+**	**+**	**+**	**+**	**+**	**+**	**+**	**+**	**+**	**+**	**+**	**+**	**+**	**+**	**+**
1 L	**+**	**+**	**+**	**+**	**+**	**+**	**+**	**+**	**+**	**+**	**+**	**+**	**+**	**+**	**+**	**+**	**+**	**+**	**-**	**-**	**-**	**+**	**+**	**+**	**+**	**+**	**+**	**+**	**+**	**+**	**+**	**+**	**+**	**+**	**+**	**+**
1 M	**+**	**+**	**+**	**+**	**+**	**+**	**+**	**+**	**+**	**+**	**+**	**+**	**+**	**+**	**+**	**+**	**+**	**+**	**-**	**-**	**-**	**+**	**+**	**+**	**+**	**+**	**+**	**+**	**+**	**+**	**+**	**+**	**+**	**+**	**+**	**+**
1 N	**+**	**+**	**+**	**+**	**+**	**+**	**+**	**+**	**+**	**+**	**+**	**+**	**+**	**+**	**+**	**+**	**+**	**+**	**+**	**+**	**+**	**+**	**+**	**+**	**+**	**+**	**+**	**+**	**+**	**+**	**+**	**+**	**+**	**+**	**+**	**+**
1O	**+**	**+**	**+**	**+**	**+**	**+**	**+**	**+**	**+**	**+**	**+**	**+**	**+**	**+**	**+**	**+**	**+**	**+**	**+**	**+**	**+**	**+**	**+**	**+**	**+**	**+**	**+**	**+**	**+**	**+**	**+**	**+**	**+**	**+**	**+**	**+**
1P	**+**	**+**	**+**	**+**	**+**	**+**	**+**	**+**	**+**	**+**	**+**	**+**	**+**	**+**	**+**	**+**	**+**	**+**	**+**	**+**	**+**	**+**	**+**	**+**	**+**	**+**	**+**	**+**	**+**	**+**	**+**	**+**	**+**	**+**	**+**	**+**
2A	**+**	**+**	**+**	**+**	**+**	**+**	**+**	**+**	**+**	**+**	**+**	**+**	**+**	**+**	**+**	**+**	**+**	**+**	**+**	**+**	**+**	**+**	**+**	**+**	**+**	**+**	**+**	**+**	**+**	**+**	**+**	**+**	**+**	**+**	**+**	**+**
2B	**+**	**+**	**+**	**+**	**+**	**+**	**+**	**+**	**+**	**+**	**+**	**+**	**+**	**+**	**+**	**+**	**+**	**+**	**+**	**+**	**+**	**+**	**+**	**+**	**+**	**+**	**+**	**+**	**+**	**+**	**+**	**+**	**+**	**+**	**+**	**+**
2C	**+**	**+**	**+**	**+**	**+**	**+**	**+**	**+**	**+**	**+**	**+**	**+**	**+**	**+**	**+**	**+**	**+**	**+**	**-**	**-**	**-**	**+**	**+**	**+**	**+**	**+**	**+**	**+**	**+**	**+**	**+**	**+**	**+**	**+**	**+**	**+**
2D	**+**	**+**	**+**	**+**	**+**	**+**	**+**	**+**	**+**	**+**	**+**	**+**	**+**	**+**	**+**	**+**	**+**	**+**	**-**	**-**	**-**	**+**	**+**	**+**	**+**	**+**	**+**	**+**	**+**	**+**	**+**	**+**	**+**	**+**	**+**	**+**
2E	**+**	**+**	**+**	**+**	**+**	**+**	**+**	**+**	**+**	**+**	**+**	**+**	**+**	**+**	**+**	**+**	**+**	**+**	**+**	**+**	**+**	**+**	**+**	**+**	**+**	**+**	**+**	**+**	**+**	**+**	**+**	**+**	**+**	**+**	**+**	**+**
2 F	**+**	**+**	**+**	**+**	**+**	**+**	**+**	**+**	**+**	**+**	**+**	**+**	**+**	**+**	**+**	**+**	**+**	**+**	**+**	**+**	**+**	**+**	**+**	**+**	**+**	**+**	**+**	**+**	**+**	**+**	**+**	**+**	**+**	**+**	**+**	**+**
2G	**+**	**+**	**+**	**+**	**+**	**+**	**+**	**+**	**+**	**+**	**+**	**+**	**+**	**+**	**+**	**+**	**+**	**+**	**+**	**+**	**+**	**+**	**+**	**+**	**+**	**+**	**+**	**+**	**+**	**+**	**+**	**+**	**+**	**+**	**+**	**+**
2H	**+**	**+**	**+**	**+**	**+**	**+**	**+**	**+**	**+**	**+**	**+**	**+**	**+**	**+**	**+**	**+**	**+**	**+**	**+**	**+**	**+**	**+**	**+**	**+**	**+**	**+**	**+**	**+**	**+**	**+**	**+**	**+**	**+**	**+**	**+**	**+**

Each of the strains were analysed at room temperature (left), 37°C (middle) and 42°C (right). Binding +; No binding -. See Additional file 1: Table S1 for full list of glycan names and structures. **1A** Galβ1-3GlcNAc; **1B** Galβ1-4GlcNAc; **1C** Galβ1-4Gal; 1D Galβ1-6GlcNAc; **1E** Galβ1-3GalNAc; **1 F** Galb1-3GalNAcβ1-4Galβ1-4Glc; **1G** Galβ1-3GlcNAcβ1-3Galβ1-4Glc; **1H** Galβ1-4GlcNAcβ1-3Galβ1-4Glc; **1I** Galβ1-4GlcNAcβ1-6(Galβ1-4GlcNAcβ1-3)Galβ1-4Glc; **1 J** Galβ1-4GlcNAcβ1-6(Galβ1-3GlcNAcβ1-3)Galβ1-4Glc; **1 K** Galα1-4Galβ1-4Glc; **1 L** GalNAcα1-O-Ser; **1 M** Galβ1-3GalNAcα1-O-Ser; **1 N** Galα1-3Gal; **1O** Galα1-3Galβ1-4GlcNAc; **1P** Galα1-3Galβ1-4Glc; **2A** Galα1-3Galβ1-4Galα1-3Gal; **2B** Galβ1-6Gal; **2C** GalNAcβ1-3Gal; **2D** GalNAcβ1-4Gal; **2E** Galα1-4Galβ1-4GlcNAc; **2 F** GalNAcα1-3Galβ1-4Glc; **2G** Galβ1-3GlcNAcβ1-3Galβ1-4GlcNAcβ1-6(Galβ1-3GlcNAcβ1-3)Galβ1-4Glc; **2H** Galβ1-3GlcNAcβ1-3Galβ1-4GlcNAcβ1-3Galβ1-4Glc.

**Table 2:** **Glucosamine and mannose binding from the glycan array analysis of twelve *C. jejuni *strains**

**Glycan ID**	**Human**																		**Chicken**																	
	**11168**			**351**			**375**			**520**			**81116**			**81–176**			**331**			**008**			**019**			**108**			**434**			**506**		
	**RT**	**37**	**42**	**RT**	**37**	**42**	**RT**	**37**	**42**	**RT**	**37**	**42**	**RT**	**37**	**42**	**RT**	**37**	**42**	**RT**	**37**	**42**	**RT**	**37**	**42**	**RT**	**37**	**42**	**RT**	**37**	**42**	**RT**	**37**	**42**	**RT**	**37**	**42**
4A	**-**	**-**	**-**	**+**	**-**	**-**	**-**	**-**	**-**	**-**	**-**	**-**	**+**	**+**	**+**	**-**	**-**	**-**	**+**	**+**	**+**	**-**	**-**	**-**	**-**	**-**	**-**	**-**	**-**	**-**	**-**	**-**	**-**	**-**	**-**	**-**
4B	**-**	**-**	**-**	**+**	**-**	**-**	**-**	**-**	**-**	**-**	**-**	**-**	**+**	**+**	**+**	**-**	**-**	**-**	**+**	**+**	**+**	**-**	**-**	**-**	**-**	**-**	**-**	**-**	**-**	**-**	**-**	**-**	**-**	**-**	**-**	**-**
4C	**-**	**-**	**-**	**+**	**-**	**-**	**-**	**-**	**-**	**-**	**-**	**-**	**+**	**+**	**+**	**-**	**-**	**-**	**-**	**-**	**-**	**+**	**+**	**+**	**-**	**-**	**-**	**-**	**-**	**-**	**-**	**-**	**-**	**+**	**+**	**+**
4D	**-**	**-**	**-**	**+**	**+**	**+**	**+**	**+**	**+**	**+**	**+**	**+**	**+**	**+**	**+**	**+**	**+**	**+**	**-**	**-**	**-**	**+**	**+**	**+**	**-**	**-**	**-**	**-**	**-**	**-**	**+**	**+**	**+**	**+**	**+**	**+**
4E	**-**	**-**	**-**	**+**	**+**	**+**	**+**	**+**	**+**	**+**	**+**	**+**	**+**	**+**	**+**	**+**	**+**	**+**	**-**	**-**	**-**	**+**	**+**	**+**	**-**	**-**	**-**	**-**	**-**	**-**	**+**	**+**	**+**	**+**	**+**	**+**
5A	**+**	**+**	**+**	**+**	**+**	**+**	**+**	**+**	**+**	**+**	**+**	**+**	**+**	**+**	**+**	**+**	**+**	**+**	**-**	**-**	**-**	**+**	**+**	**+**	**+**	**+**	**+**	**+**	**+**	**+**	**+**	**+**	**+**	**+**	**+**	**+**
5B	**+**	**+**	**+**	**+**	**+**	**+**	**+**	**+**	**+**	**+**	**+**	**+**	**+**	**+**	**+**	**+**	**+**	**+**	**-**	**-**	**-**	**+**	**+**	**+**	**+**	**+**	**+**	**+**	**+**	**+**	**+**	**+**	**+**	**+**	**+**	**+**
5C	**-**	**-**	**-**	**+**	**-**	**-**	**+**	**-**	**-**	**+**	**-**	**-**	**+**	**+**	**+**	**+**	**-**	**-**	**+**	**+**	**+**	**+**	**-**	**-**	**+**	**-**	**-**	**+**	**-**	**-**	**-**	**-**	**-**	**-**	**-**	**-**
5D	**-**	**-**	**-**	**+**	**-**	**-**	**+**	**-**	**-**	**+**	**-**	**-**	**+**	**+**	**+**	**+**	**-**	**-**	**-**	**-**	**-**	**+**	**-**	**-**	**+**	**-**	**-**	**+**	**-**	**-**	**-**	**-**	**-**	**-**	**-**	**-**
5E	**+**	**-**	**-**	**+**	**-**	**-**	**+**	**-**	**-**	**+**	**-**	**-**	**+**	**+**	**+**	**+**	**-**	**-**	**+**	**+**	**+**	**+**	**-**	**-**	**+**	**-**	**-**	**+**	**-**	**-**	**+**	**-**	**-**	**+**	**-**	**-**
5 F	**+**	**-**	**-**	**+**	**-**	**-**	**+**	**-**	**-**	**+**	**-**	**-**	**+**	**+**	**+**	**+**	**-**	**-**	**+**	**+**	**+**	**+**	**-**	**-**	**+**	**-**	**-**	**+**	**-**	**-**	**+**	**-**	**-**	**+**	**-**	**-**
5G	**+**	**-**	**-**	**+**	**+**	**+**	**+**	**-**	**-**	**+**	**-**	**-**	**+**	**+**	**+**	**+**	**-**	**-**	**+**	**+**	**+**	**+**	**-**	**-**	**+**	**-**	**-**	**+**	**+**	**+**	**+**	**+**	**+**	**+**	**+**	**+**
5H	**+**	**-**	**-**	**+**	**+**	**+**	**+**	**-**	**-**	**+**	**-**	**-**	**+**	**+**	**+**	**+**	**-**	**-**	**+**	**+**	**+**	**+**	**-**	**-**	**+**	**-**	**-**	**+**	**+**	**+**	**+**	**+**	**+**	**+**	**+**	**+**

Each of the strains were analysed at room temperature (left), 37°C (middle) and 42°C (right). Binding +; No binding -. 4A-4D are repeating N-Acetylglucosamine (GlcNAc) structures that increase in length from A-D (**4A** GlcNAcβ1-4GlcNAc; **4B** GlcNAcβ1-4GlcNAcβ1-4GlcNAc; **4C** GlcNAcβ1-4GlcNAcβ1-4GlcNAcβ1-4GlcNAc; **4D** GlcNAcβ1-4GlcNAcβ1-4GlcNAcβ1-4GlcNAcβ1-4GlcNAcβ1-4GlcNAc; **4E** GlcNAcβ1-4MurNAc). 5A and B are mannose containing GlcNAc structures (**5A** GlcNAcβ1-2Man; 5B GlcNAcβ1-2Manα1-6(GlcNAcβ1-2Manα1-3)Man), while 5C-5H are mannose of differing linkages and branch size (5C Manα1-2Man; 5D Manα1-3Man; 5E Manα1-4Man; 5 F Manα1-6Man; 5G Manα1-6(Manα1-3)Man; 5H Manα1-6(Manα1-3)Manα1-6(Manα1-3)Man). Please see Additional file 1: Table S1 for full list of glycan names and structures).

**Table 3:** **Binding of sialylated structures from the glycan array analysis of twelve *C. jejuni *strains**

**Glycan ID**	**Human**																		**Chicken**																	
	**11168**			**351**			**375**			**520**			**81116**			**81–176**			**331**			**008**			**019**			**108**			**434**			**506**		
	**RT**	**37**	**42**	**RT**	**37**	**42**	**RT**	**37**	**42**	**RT**	**37**	**42**	**RT**	**37**	**42**	**RT**	**37**	**42**	**RT**	**37**	**42**	**RT**	**37**	**42**	**RT**	**37**	**42**	**RT**	**37**	**42**	**RT**	**37**	**42**	**RT**	**37**	**42**
10A	**+**	**-**	**-**	**+**	**+**	**+**	**+**	**+**	**+**	**+**	**+**	**+**	**+**	**-**	**-**	**+**	**-**	**-**	**+**	**+**	**+**	**+**	**-**	**-**	**+**	**-**	**-**	**+**	**-**	**-**	**+**	**+**	**+**	**+**	**+**	**+**
10B	**+**	**-**	**-**	**+**	**+**	**+**	**+**	**+**	**+**	**+**	**+**	**+**	**+**	**-**	**-**	**+**	**-**	**-**	**+**	**+**	**+**	**+**	**-**	**-**	**+**	**-**	**-**	**+**	**-**	**-**	**+**	**+**	**+**	**+**	**+**	**+**
10C	**+**	**-**	**-**	**+**	**-**	**-**	**+**	**-**	**-**	**+**	**-**	**-**	**+**	**+**	**+**	**+**	**-**	**-**	**+**	**-**	**-**	**+**	**-**	**-**	**+**	**-**	**-**	**+**	**-**	**-**	**+**	**-**	**-**	**+**	**-**	**-**
10D	**+**	**+**	**+**	**+**	**+**	**+**	**+**	**+**	**+**	**+**	**+**	**+**	**+**	**+**	**+**	**+**	**+**	**+**	**+**	**+**	**+**	**+**	**+**	**+**	**+**	**+**	**+**	**+**	**+**	**+**	**+**	**+**	**+**	**+**	**+**	**+**
10 K	**+**	**-**	**-**	**+**	**-**	**-**	**+**	**-**	**-**	**+**	**-**	**-**	**+**	**+**	**+**	**+**	**-**	**-**	**+**	**-**	**-**	**+**	**-**	**-**	**+**	**-**	**-**	**+**	**-**	**-**	**+**	**-**	**-**	**+**	**-**	**-**
10 L	**+**	**-**	**-**	**+**	**-**	**-**	**+**	**-**	**-**	**+**	**-**	**-**	**+**	**-**	**-**	**+**	**-**	**-**	**+**	**-**	**-**	**+**	**-**	**-**	**+**	**-**	**-**	**+**	**-**	**-**	**+**	**-**	**-**	**+**	**-**	**-**
10 M	**+**	**-**	**-**	**+**	**-**	**-**	**+**	**-**	**-**	**+**	**-**	**-**	**+**	**-**	**-**	**+**	**-**	**-**	**+**	**-**	**-**	**+**	**-**	**-**	**+**	**-**	**-**	**+**	**-**	**-**	**+**	**-**	**-**	**+**	**-**	**-**
10 N	**+**	**-**	**-**	**+**	**-**	**-**	**+**	**-**	**-**	**+**	**-**	**-**	**+**	**-**	**-**	**+**	**-**	**-**	**+**	**-**	**-**	**+**	**-**	**-**	**+**	**-**	**-**	**+**	**-**	**-**	**+**	**-**	**-**	**+**	**-**	**-**
10O	**+**	**-**	**-**	**+**	**-**	**-**	**+**	**-**	**-**	**+**	**-**	**-**	**+**	**-**	**-**	**+**	**-**	**-**	**+**	**-**	**-**	**+**	**-**	**-**	**+**	**-**	**-**	**+**	**-**	**-**	**+**	**-**	**-**	**+**	**-**	**-**
10P	**+**	**-**	**-**	**+**	**-**	**-**	**+**	**-**	**-**	**+**	**-**	**-**	**+**	**-**	**-**	**+**	**-**	**-**	**+**	**-**	**-**	**+**	**-**	**-**	**+**	**-**	**-**	**+**	**-**	**-**	**+**	**-**	**-**	**+**	**-**	**-**
11A	**+**	**-**	**-**	**+**	**-**	**-**	**+**	**-**	**-**	**+**	**-**	**-**	**+**	**-**	**-**	**+**	**-**	**-**	**+**	**-**	**-**	**+**	**-**	**-**	**+**	**-**	**-**	**+**	**-**	**-**	**+**	**-**	**-**	**+**	**-**	**-**
11B	**+**	**-**	**-**	**+**	**-**	**-**	**+**	**-**	**-**	**+**	**-**	**-**	**+**	**-**	**-**	**+**	**-**	**-**	**+**	**-**	**-**	**+**	**-**	**-**	**+**	**-**	**-**	**+**	**-**	**-**	**+**	**-**	**-**	**+**	**-**	**-**
11C	**+**	**-**	**-**	**+**	**-**	**-**	**+**	**-**	**-**	**+**	**-**	**-**	**+**	**-**	**-**	**+**	**-**	**-**	**+**	**-**	**-**	**+**	**-**	**-**	**+**	**-**	**-**	**+**	**-**	**-**	**+**	**-**	**-**	**+**	**-**	**-**
11D	**+**	**-**	**-**	**+**	**-**	**-**	**+**	**-**	**-**	**+**	**+**	**+**	**+**	**-**	**-**	**+**	**-**	**-**	**+**	**-**	**-**	**+**	**-**	**-**	**+**	**-**	**-**	**+**	**-**	**-**	**+**	**-**	**-**	**+**	**-**	**-**

Each of the strains were analysed at room temperature (left), 37°C (middle) and 42°C (right). Binding +; No binding -. See Additional file 1: Table S1 for full list and structures of glycans. 10A Neu5Acα2-3Galβ1-3(Fucα1-4)GlcNAc; 10B Neu5Acα2-3Galβ1-4(Fucα1-3)GlcNAc; 10C Neu5Acα2-3Galβ1-3GlcNAcβ1-3Galβ1-4Glc; 10D Galβ1-4(Fucα1-3)GlcNAcβ1-6(Neu5Acα2-6Galβ1-4GlcNAcβ1-3)Galβ1-4Glc; 10 K Neu5Acα2-3Galβ1-4GlcNAc; 10 L Neu5Acα2-6Galβ1-4GlcNAc; 10 M Neu5Acα2-3Galβ1-3GlcNAcβ1-3Galβ1-4Glc; 10 N Galβ1-3(Neu5Acα2-6)GlcNAcβ1-3Galβ1-4Glc; 10O Neu5Acα2-6Galβ1-4GlcNAcβ1-3Galβ1-4Glc; 10P Neu5Acα2-3Galβ1-3(Neu5Acα2-6)GlcNAcβ1-3Galβ1-4Glc; 11A Neu5Acα2-3Galβ1-4Glc; 11B Neu5Acα2-6Galβ1-4Glc; 11C (Neu5Acα2-8Neu5Ac)n (n < 50); 11D Neu5Acα2-6Galβ1-4GlcNAcβ1-2Manα1-6(Neu5Acα2-6Galβ1-4GlcNAcβ1-2Manα1-6)Manβ1-4GlcNAcβ1-4GlcNAc-Asn.

**Table 4:** **Binding of GAG and GAG related structures from the glycan array analysis of twelve *C. jejuni *strains**

**Glycan ID**	**Human**																		**Chicken**																	
	**11168**			**351**			**375**			**520**			**81116**			**81–176**			**331**			**008**			**019**			**108**			**434**			**506**		
	**RT**	**37**	**42**	**RT**	**37**	**42**	**RT**	**37**	**42**	**RT**	**37**	**42**	**RT**	**37**	**42**	**RT**	**37**	**42**	**RT**	**37**	**42**	**RT**	**37**	**42**	**RT**	**37**	**42**	**RT**	**37**	**42**	**RT**	**37**	**42**	**RT**	**37**	**42**
12A	**+**	**+**	**+**	**-**	**-**	**-**	**-**	**-**	**-**	**-**	**-**	**-**	**+**	**+**	**+**	**-**	**-**	**-**	**-**	**-**	**-**	**+**	**+**	**+**	**+**	**+**	**+**	**+**	**+**	**+**	**+**	**+**	**+**	**+**	**+**	**+**
12B	**+**	**+**	**+**	**-**	**-**	**-**	**-**	**-**	**-**	**-**	**-**	**-**	**+**	**+**	**+**	**-**	**-**	**-**	**-**	**-**	**-**	**+**	**+**	**+**	**+**	**+**	**+**	**+**	**+**	**+**	**+**	**+**	**+**	**+**	**+**	**+**
12C	**+**	**+**	**+**	**-**	**-**	**-**	**-**	**-**	**-**	**-**	**-**	**-**	**+**	**+**	**+**	**-**	**-**	**-**	**-**	**-**	**-**	**+**	**+**	**+**	**+**	**+**	**+**	**+**	**+**	**+**	**+**	**+**	**+**	**+**	**+**	**+**
12D	**+**	**+**	**+**	**-**	**-**	**-**	**-**	**-**	**-**	**-**	**-**	**-**	**+**	**+**	**+**	**-**	**-**	**-**	**-**	**-**	**-**	**+**	**+**	**+**	**+**	**+**	**+**	**+**	**+**	**+**	**+**	**+**	**+**	**+**	**+**	**+**
12E	**+**	**+**	**+**	**-**	**-**	**-**	**-**	**-**	**-**	**-**	**-**	**-**	**+**	**+**	**+**	**-**	**-**	**-**	**-**	**-**	**-**	**+**	**+**	**+**	**+**	**+**	**+**	**+**	**+**	**+**	**+**	**+**	**+**	**+**	**+**	**+**
12 F	**+**	**+**	**+**	**-**	**-**	**-**	**-**	**-**	**-**	**-**	**-**	**-**	**+**	**+**	**+**	**-**	**-**	**-**	**-**	**-**	**-**	**+**	**+**	**+**	**+**	**+**	**+**	**+**	**+**	**+**	**+**	**+**	**+**	**+**	**+**	**+**
12G	**-**	**-**	**-**	**-**	**-**	**-**	**-**	**-**	**-**	**-**	**-**	**-**	**+**	**+**	**+**	**-**	**-**	**-**	**-**	**-**	**-**	**-**	**-**	**-**	**-**	**-**	**-**	**-**	**-**	**-**	**-**	**-**	**-**	**-**	**-**	**-**
12H	**-**	**-**	**-**	**-**	**-**	**-**	**-**	**-**	**-**	**-**	**-**	**-**	**+**	**+**	**+**	**-**	**-**	**-**	**-**	**-**	**-**	**-**	**-**	**-**	**-**	**-**	**-**	**-**	**-**	**-**	**-**	**-**	**-**	**-**	**-**	**-**
12I	**-**	**-**	**-**	**-**	**-**	**-**	**-**	**-**	**-**	**-**	**-**	**-**	**+**	**+**	**+**	**-**	**-**	**-**	**-**	**-**	**-**	**-**	**-**	**-**	**-**	**-**	**-**	**-**	**-**	**-**	**-**	**-**	**-**	**-**	**-**	**-**
12 J	**-**	**-**	**-**	**-**	**-**	**-**	**-**	**-**	**-**	**-**	**-**	**-**	**+**	**+**	**+**	**-**	**-**	**-**	**-**	**-**	**-**	**-**	**-**	**-**	**-**	**-**	**-**	**-**	**-**	**-**	**-**	**-**	**-**	**-**	**-**	**-**
12 K	**-**	**-**	**-**	**-**	**-**	**-**	**-**	**-**	**-**	**-**	**-**	**-**	**+**	**+**	**+**	**-**	**-**	**-**	**-**	**-**	**-**	**-**	**-**	**-**	**-**	**-**	**-**	**-**	**-**	**-**	**-**	**-**	**-**	**-**	**-**	**-**
12 L	**-**	**-**	**-**	**-**	**-**	**-**	**-**	**-**	**-**	**-**	**-**	**-**	**+**	**+**	**+**	**-**	**-**	**-**	**-**	**-**	**-**	**-**	**-**	**-**	**-**	**-**	**-**	**-**	**-**	**-**	**-**	**-**	**-**	**-**	**-**	**-**
12 M	**-**	**-**	**-**	**-**	**-**	**-**	**-**	**-**	**-**	**-**	**-**	**-**	**+**	**+**	**+**	**-**	**-**	**-**	**-**	**-**	**-**	**-**	**-**	**-**	**-**	**-**	**-**	**-**	**-**	**-**	**-**	**-**	**-**	**-**	**-**	**-**
12 N	**-**	**-**	**-**	**-**	**-**	**-**	**-**	**-**	**-**	**-**	**-**	**-**	**+**	**+**	**+**	**-**	**-**	**-**	**-**	**-**	**-**	**-**	**-**	**-**	**-**	**-**	**-**	**-**	**-**	**-**	**-**	**-**	**-**	**-**	**-**	**-**
12O	**-**	**-**	**-**	**-**	**-**	**-**	**-**	**-**	**-**	**-**	**-**	**-**	**+**	**+**	**+**	**-**	**-**	**-**	**-**	**-**	**-**	**-**	**-**	**-**	**-**	**-**	**-**	**-**	**-**	**-**	**-**	**-**	**-**	**-**	**-**	**-**
12P	**-**	**-**	**-**	**-**	**-**	**-**	**-**	**-**	**-**	**-**	**-**	**-**	**+**	**+**	**+**	**-**	**-**	**-**	**-**	**-**	**-**	**-**	**-**	**-**	**-**	**-**	**-**	**-**	**-**	**-**	**-**	**-**	**-**	**-**	**-**	**-**
13A	**-**	**-**	**-**	**-**	**-**	**-**	**-**	**-**	**-**	**+**	**+**	**+**	**+**	**+**	**+**	**-**	**-**	**-**	**-**	**-**	**-**	**-**	**-**	**-**	**-**	**-**	**-**	**-**	**-**	**-**	**-**	**-**	**-**	**-**	**-**	**-**
13B	**-**	**-**	**-**	**-**	**-**	**-**	**-**	**-**	**-**	**-**	**-**	**-**	**+**	**+**	**+**	**-**	**-**	**-**	**-**	**-**	**-**	**-**	**-**	**-**	**-**	**-**	**-**	**-**	**-**	**-**	**-**	**-**	**-**	**-**	**-**	**-**
13C	**-**	**-**	**-**	**-**	**-**	**-**	**-**	**-**	**-**	**-**	**-**	**-**	**+**	**+**	**+**	**-**	**-**	**-**	**-**	**-**	**-**	**-**	**-**	**-**	**-**	**-**	**-**	**-**	**-**	**-**	**-**	**-**	**-**	**-**	**-**	**-**
13D	**-**	**-**	**-**	**-**	**-**	**-**	**-**	**-**	**-**	**-**	**-**	**-**	**+**	**+**	**+**	**-**	**-**	**-**	**-**	**-**	**-**	**-**	**-**	**-**	**-**	**-**	**-**	**-**	**-**	**-**	**-**	**-**	**-**	**-**	**-**	**-**
13E	**-**	**-**	**-**	**-**	**-**	**-**	**-**	**-**	**-**	**+**	**+**	**+**	**+**	**+**	**+**	**-**	**-**	**-**	**+**	**+**	**+**	**-**	**-**	**-**	**-**	**-**	**-**	**-**	**-**	**-**	**-**	**-**	**-**	**-**	**-**	**-**
13 F	**+**	**+**	**+**	**+**	**+**	**+**	**+**	**+**	**+**	**-**	**-**	**-**	**+**	**+**	**+**	**+**	**+**	**+**	**-**	**-**	**-**	**+**	**+**	**+**	**-**	**-**	**-**	**+**	**+**	**+**	**+**	**+**	**+**	**-**	**-**	**-**
13G	**+**	**+**	**+**	**+**	**+**	**+**	**+**	**+**	**+**	**+**	**+**	**+**	**+**	**+**	**+**	**+**	**+**	**+**	**+**	**+**	**+**	**+**	**+**	**+**	**+**	**+**	**+**	**+**	**+**	**+**	**+**	**+**	**+**	**+**	**+**	**+**
13H	**+**	**+**	**+**	**+**	**+**	**+**	**+**	**+**	**+**	**+**	**+**	**+**	**+**	**+**	**+**	**+**	**+**	**+**	**+**	**+**	**+**	**+**	**+**	**+**	**+**	**+**	**+**	**+**	**+**	**+**	**+**	**+**	**+**	**+**	**+**	**+**
13I	**+**	**+**	**+**	**+**	**+**	**+**	**+**	**+**	**+**	**+**	**+**	**+**	**+**	**+**	**+**	**+**	**+**	**+**	**+**	**+**	**+**	**+**	**+**	**+**	**+**	**+**	**+**	**+**	**+**	**+**	**+**	**+**	**+**	**+**	**+**	**+**
13 J	**+**	**+**	**+**	**+**	**+**	**+**	**+**	**+**	**+**	**+**	**+**	**+**	**+**	**+**	**+**	**+**	**+**	**+**	**+**	**+**	**+**	**+**	**+**	**+**	**+**	**+**	**+**	**+**	**+**	**+**	**+**	**+**	**+**	**+**	**+**	**+**
13 K	**+**	**+**	**+**	**+**	**+**	**+**	**+**	**+**	**+**	**+**	**+**	**+**	**+**	**+**	**+**	**+**	**+**	**+**	**+**	**+**	**+**	**+**	**+**	**+**	**+**	**+**	**+**	**+**	**+**	**+**	**+**	**+**	**+**	**+**	**+**	**+**
13 L	**+**	**+**	**+**	**+**	**+**	**+**	**+**	**+**	**+**	**+**	**+**	**+**	**+**	**+**	**+**	**+**	**+**	**+**	**+**	**+**	**+**	**+**	**+**	**+**	**+**	**+**	**+**	**+**	**+**	**+**	**+**	**+**	**+**	**+**	**+**	**+**
13 M	**+**	**+**	**+**	**+**	**+**	**+**	**+**	**+**	**+**	**+**	**+**	**+**	**+**	**+**	**+**	**+**	**+**	**+**	**+**	**+**	**+**	**+**	**+**	**+**	**+**	**+**	**+**	**+**	**+**	**+**	**+**	**+**	**+**	**+**	**+**	**+**
13 N	**+**	**+**	**+**	**+**	**+**	**+**	**+**	**+**	**+**	**+**	**+**	**+**	**+**	**+**	**+**	**+**	**+**	**+**	**+**	**+**	**+**	**+**	**+**	**+**	**+**	**+**	**+**	**+**	**+**	**+**	**+**	**+**	**+**	**+**	**+**	**+**
13O	**+**	**+**	**+**	**+**	**+**	**+**	**+**	**+**	**+**	**+**	**+**	**+**	**+**	**+**	**+**	**+**	**+**	**+**	**+**	**+**	**+**	**+**	**+**	**+**	**+**	**+**	**+**	**+**	**+**	**+**	**+**	**+**	**+**	**+**	**+**	**+**
13P	**+**	**+**	**+**	**+**	**+**	**+**	**+**	**+**	**+**	**+**	**+**	**+**	**+**	**+**	**+**	**+**	**+**	**+**	**+**	**+**	**+**	**+**	**+**	**+**	**+**	**+**	**+**	**+**	**+**	**+**	**+**	**+**	**+**	**+**	**+**	**+**
14A	**+**	**+**	**+**	**+**	**+**	**+**	**+**	**+**	**+**	**+**	**+**	**+**	**+**	**+**	**+**	**+**	**+**	**+**	**+**	**+**	**+**	**+**	**+**	**+**	**+**	**+**	**+**	**+**	**+**	**+**	**+**	**+**	**+**	**+**	**+**	**+**
14B	**+**	**+**	**+**	**+**	**+**	**+**	**+**	**+**	**+**	**+**	**+**	**+**	**+**	**+**	**+**	**+**	**+**	**+**	**+**	**+**	**+**	**+**	**+**	**+**	**+**	**+**	**+**	**+**	**+**	**+**	**+**	**+**	**+**	**+**	**+**	**+**
14C	**+**	**+**	**+**	**+**	**+**	**+**	**+**	**+**	**+**	**+**	**+**	**+**	**+**	**+**	**+**	**+**	**+**	**+**	**+**	**+**	**+**	**+**	**+**	**+**	**+**	**+**	**+**	**+**	**+**	**+**	**+**	**+**	**+**	**+**	**+**	**+**
14D	**+**	**+**	**+**	**+**	**+**	**+**	**+**	**+**	**+**	**+**	**+**	**+**	**+**	**+**	**+**	**+**	**+**	**+**	**+**	**+**	**+**	**+**	**+**	**+**	**+**	**+**	**+**	**+**	**+**	**+**	**+**	**+**	**+**	**+**	**+**	**+**
14E	**+**	**+**	**+**	**+**	**+**	**+**	**+**	**+**	**+**	**+**	**+**	**+**	**+**	**+**	**+**	**+**	**+**	**+**	**+**	**+**	**+**	**+**	**+**	**+**	**+**	**+**	**+**	**+**	**+**	**+**	**+**	**+**	**+**	**+**	**+**	**+**
14 F	**+**	**+**	**+**	**+**	**+**	**+**	**+**	**+**	**+**	**+**	**+**	**+**	**+**	**+**	**+**	**+**	**+**	**+**	**+**	**+**	**+**	**+**	**+**	**+**	**+**	**+**	**+**	**+**	**+**	**+**	**+**	**+**	**+**	**+**	**+**	**+**
14G	**+**	**+**	**+**	**+**	**+**	**+**	**+**	**+**	**+**	**+**	**+**	**+**	**+**	**+**	**+**	**+**	**+**	**+**	**+**	**+**	**+**	**+**	**+**	**+**	**+**	**+**	**+**	**+**	**+**	**+**	**+**	**+**	**+**	**+**	**+**	**+**
14H	**+**	**+**	**+**	**+**	**+**	**+**	**+**	**+**	**+**	**+**	**+**	**+**	**+**	**+**	**+**	**+**	**+**	**+**	**+**	**+**	**+**	**+**	**+**	**+**	**+**	**+**	**+**	**+**	**+**	**+**	**+**	**+**	**+**	**+**	**+**	**+**
14I	**+**	**+**	**+**	**+**	**+**	**+**	**+**	**+**	**+**	**+**	**+**	**+**	**+**	**+**	**+**	**+**	**+**	**+**	**+**	**+**	**+**	**+**	**+**	**+**	**+**	**+**	**+**	**+**	**+**	**+**	**+**	**+**	**+**	**+**	**+**	**+**

Each of the strains were analysed at room temperature (left), 37°C (middle) and 42°C (right). Binding +; No binding -. 12A-12 F are Carrageenan repeats; 12G-13E are digested Glycoaminoglycans in their most basic non-repeating unit (12G ΔUA-2S **→** GlcNS-6S Na4 (I-S); 12H ΔUA **→** GlucNS-6S Na3 (II-S); 12I ΔUA **→** 2S-GlcNS Na3 (III-S); 12 J ΔUA **→** 2S-GlcNAc-6S Na3 (I-A); 12 K ΔUA **→** GlcNAc-6S Na2 (II-A); 12 L ΔUA **→** 2S-GlcNAc Na2 (III-A); 12 M ΔUA **→** GlcNAc Na (IV-A); 12 N ΔUA **→** GalNAc-4S Na2 (ΔDi-4S); 12O ΔUA **→** GalNAc-6S Na2 (ΔDi-6S); 12P ΔUA **→** GalNAc-4S,6S Na3 (ΔDi-disE); 13A ΔUA **→** 2S-GalNAc-4S Na2 (ΔDi-disB); 13B ΔUA **→** 2S-GalNAc-6S Na3 (ΔDi-disD); 13C ΔUA **→** 2S-GalNAc-4S-6S Na4 (ΔDi-tisS); 13D ΔUA **→** 2S-GalNAc-6S Na2 (ΔDi-UA2S); 13E ΔUA **→** GlcNAc Na (ΔDi-HA); 13 F-14I are larger repeating Glycoaminoglycans ranging from 4mers to 1.6MDa in size (13 F (GlcAβ1-3GlcNAcβ1-4)n (n = 4); 13G (GlcAβ1-3GlcNAcβ1-4)n (n = 8); 13H. (GlcAβ1-3GlcNAcβ1-4)n (n = 10); 13I (GlcAβ1-3GlcNAcβ1-4)n (n = 12); 13 J (GlcA/IdoAα/β1-4GlcNAcα1-4)n (n = 200); 13 K (GlcA/IdoAβ1-3(±4/6S)GalNAcβ1-4)n (n < 250); 13 L ((±2S)GlcA/IdoAα/b1-3(±4S)GalNAcβ1-4)n (n < 250);13 M (GlcA/IdoAβ1-3(±6S)GalNAcβ1-4)n (n < 250); 13 N (GlcAβ1-3GlcNAcβ1-4)n (n = 4); 130 (GlcAβ1-3GlcNAcβ1-4)n (n = 6); 13P (GlcAβ1-3GlcNAcβ1-4)n (n = 8); 14A (GlcAβ1-3GlcNAcβ1-4)n (n = 10); 14B (GlcAβ1-3GlcNAcβ1-4)n (n = 12); 14C (GlcAβ1-3GlcNAcβ1-4)n (n = 14); 14D(GlcAβ1-3GlcNAcβ1-4)n (n = 16); 14E (GlcAβ1-3GlcNAcβ1-4)n (30000 Da); 14 F (GlcAβ1-3GlcNAcβ1-4)n (107000 Da); 14G (GlcAβ1-3GlcNAcβ1-4)n (190000 Da); 14H (GlcAβ1-3GlcNAcβ1-4)n (220000 Da); 14I (GlcAβ1-3GlcNAcβ1-4)n (1600000 Da); 14 J (GlcA/IdoAα/ IdoASα/β1-4GlcNAc/GlcNS/GlcNAc6Sα1-4)n; 14 K (Glcβ1-4Glc)n; see Additional file 1: Table S1 for full list of glycan names and structures).

All but two of the strains, *C. jejuni* 331 and 520, bound all galactose structures present on the array (Table 1). The chicken isolate *C. jejuni* 331 recognised the least number of terminal galactose structures only recognising 15 of the 24 printed structures. Of the nine terminal galactose structures that *C. jejuni* 331 fails to recognise, seven are disaccharides and no binding was observed to disaccharides containing GalNAc residues. Human isolate *C. jejuni* 520 failed to bind two structures; one was asialo-GM1 (1 F) and a terminal α-1-4 linked galactose (1 K), both these structures offer unique terminal glycans, with no other glycan present on the array presenting the same structure on the reducing end (Table 1).

Most variability was observed in binding to N-acetylglucosamine (Table 2; 4A-4E), mannosylated (Table 2; 5A-5H) and sialylated (Table 3; 10A-11D) glycans, with different strains recognising variable subsets of each of these structures. Binding to mannose and sialic acid was consistently growth condition dependent for the majority of strains tested (10/12) with differential binding occurring depending on whether the strains were grown under conditions mimicking different hosts (37/42°C with microaerobic conditions) or environmentally exposed (room temperature with normal oxygen; Tables 2 and 3).

Chitin structures (GlcNAc_n_; Table 2, 4A-4D) are present on the array as a variable repeat length glycan (2–5 sugars in length), with the recognition of these repeat lengths differing between strains tested. The non-invasive chicken isolate 331 has a preference for the smaller repeats (GlcNAc_2-3_; Table 2, 4A and B), while almost all other strains preferentially bound to the larger fragments (GlcNAc_5_; Table 2, 4D). *C. jejuni* 11168 was found not to bind any of these structures.

Though sialic acid was in general only recognised under conditions mimicking environmental stress there were several sialylated structures that were also recognised by all *C. jejuni* strains grow under host-like conditions. Typically the sialylated structures recognised by *C. jejuni* grown under host-like conditions were also fucosylated. The most noteworthy was binding of the sialylated and fucosylated structures, SialylLewis A and X (Table 3, 10A and B). Binding differences were observed for human isolates 351, 375 and 520 and chicken isolates 331, 434 and 506, however, these differences could not be attributed to a specific host, chicken or human. Also, *C. jejuni* strains 520 (human), 81116 (human) and 019 (chicken) were shown to bind at least one non-fucoslylated sialic acid containing structure when grown under host-like conditions. For *C. jejuni* 520 and 019 this structure is a complex, branched, *N*-linked glycan that contains within its 11 residues; a mixture of sialic acid (terminal positions on the branches), galactose, mannose and glucosamine linked directly to an asparagine. Therefore, the binding of sialic acid by *C. jejuni* 520 and 019 to this structure may not be due to any specific recognition of sialic acid under host-like growth conditions.

All *C. jejuni* strains widely recognised structures containing fucose including the bianternary structure present in the sialylated glycans (Table 3; 10D), with no significant difference observed between the twelve strains (data not shown; see Additional file 1: Table S1 for list of structures tested).

Numerous differences were observed for the binding of glycoaminoglycans (GAGs) and related structures between the *C. jejuni* strains tested (Table 4). Recognition of GAG structures has not previously been reported for *C. jejuni*. We found that carageenan structures (red seaweed extract with structural similarities to GAGs) were preferred by chicken isolates, with five of the six isolates recognising these structures. Only *C. jejuni* 331 did not bind to these structures (Table 4; 12A-F). Of the human isolates, only *C. jejuni* 11168 and 81116 bound to the carageenan structures. *C. jejuni* 81116 was the only strain that bound with any consistency to the enzymatically digested GAG disaccharide fragments (Table 4; 12G-13H). However, all strains of *C. jejuni* tested bound to hyaluronin, chondrotin, heparin and dermatin.

### Lectin and glycan competition adherence assays

As previously shown with *C. jejuni* 11168, lectins that recognise structures similar or identical to those recognised by *C. jejuni,* can be used to inhibit adherence to the surface of Caco-2 cells [3]. For the adherence inhibition assays, using both lectins and free glycans, *C. jejuni* was grown at 37°C in a microaerobic environment, mimicking one of the growth conditions used in glycan arrays assays. Two lectins were tested; ConA (mannose binding lectin) and UEA-I (fucose binding lectin).

**Figure 1:**
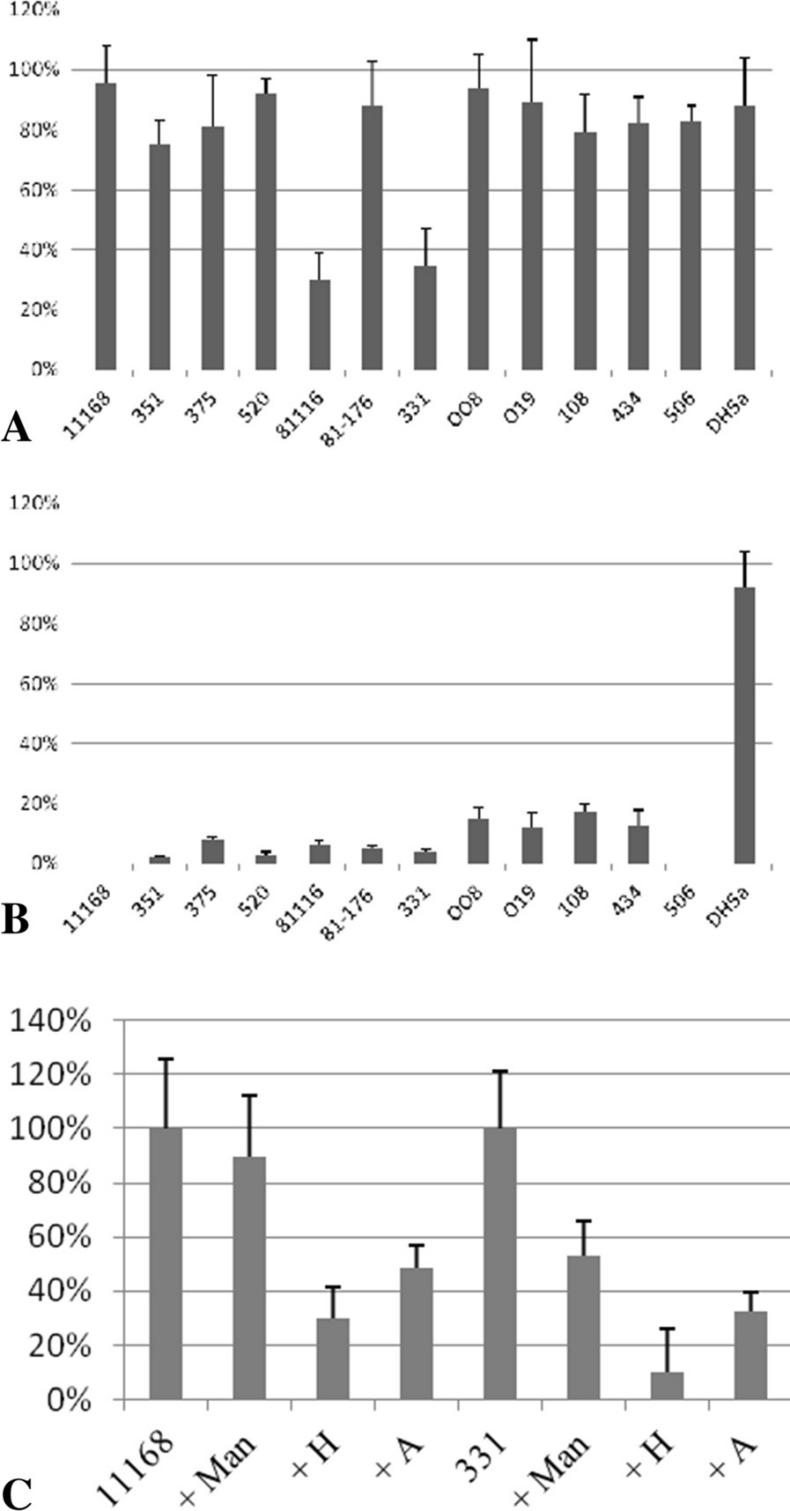
**Lectin and free glycan competition assays.** Comparison between normal adherence (100%) and inhibition with lectin or glycan pre-treatment. The smaller the bar the less *C. jejuni* adhered in the presence of the lectin/glycan. **A.** ConA competition of *C. jejuni* adherence to Caco-2 cells; **B.** UEA-I competition of *C. jejuni* adherence to Caco-2 cells. **C.** Competion assays with free glycans with *C. jejuni* 11168 and 331 adhering to Caco-2 cells.

As predicted from the array results, ConA had the greatest inhibitory effects on the adherence of *C. jejuni* 81116 and 331 with reductions of more than 70%, no significant difference was observed for the other strains tested (Figure 1A). UEA-I resulted in significant reduction in adherence for all strains tested but did not affect the adherence of the control *E. coli* DH5a strain (Figure 1B).

Free glycans were also tested on the adherence of two *C. jejuni* strains; the clinical isolate 11168 and the chicken isolate 331. Using 100 μM of free blood group antigens, A blood group trisaccharide (glycan 7 K on the array) and the H disaccharide (O-blood group antigen; glycan 7 F on the array), resulted in the significant decrease of adherence of both *C. jejuni* 11168 (P < 0.05) and 331 (P < 0.05) to Caco-2 cells (Figure 1C). Free mannose (α1-2 Mannobiose at 100 μM; glycan 5C on the array) had no effect on the binding of *C. jejuni* 11168 to Caco-2 cells but did significantly reduce the adherence of *C. jejuni* 331 (P < 0.05; Figure 1C). This result is in agreement with the array data, with both strains binding blood group antigens but only *C. jejuni* 331 recognising mannose under the condition tested (Table 2).

## Discussion

All *C. jejuni* strains tested in this study showed remarkable similarity for the general types of glycan structures that were recognised. Looking globally at the total array, *C. jejuni* behaves as a species with little variation, each strain bound to both α and β galactose, terminal and subterminal fucosylated structures and to a subset of glycoaminoglycans at all conditions tested. All strains also exhibited binding to a broader range of glycans when placed under environmental stress. Only chitin, a common insect and crustacean glycan, showed major differences when viewed from a global perspective, with one strain, *C. jejuni* 11168, failing to recognise any chitin molecule. No major difference was observed between *C. jejuni* strains isolated from different hosts.

The possibility of galactose and fucose being involved in the persistent colonisation of *C. jejuni*[3,4] is supported by the interactions observed in this study. All twelve strains, whether isolated from avian or clinical sources, bound broadly to uncapped galactose structures and fucosylated structures. These results were confirmed by inhibition of adherence to cells blocked by competing *C. jejuni* adherence with UEA-I.

Of the strains tested only one chicken isolate (331) and one clinical isolate (520) showed variability in the galactose structures bound. Of interest is the broad specificity of all the *C. jejuni* strains for galactose and fucosylated structures. Only strain, *C. jejuni* 520, showed binding differences based on linkage specificity with Galβ1-3GalNAc (asialo-GM1 1 F) and terminal α-1-4 linked di-galactose (1 K) glycan structures not being recognised.

The fact that *C. jejuni* recognises a broad range of both α and β linked galactose may offer some explanation for such a broad host range, as might the lack of specificity for linkage and position of fucose in fucosylated structures. α-linked galactose are not common in humans but are common in many other mammals and avian species [13-17]. Some strains of *C. jejuni* are known to produce the P-antigen, a terminal α-linked galactose, as a part of their LOS structure to mimic the glycans of potential avian and non-human mammalian hosts [13,18]. β-linked galactose structures are common to all animals known to be infected with *C. jejuni*. The fact that *C. jejuni* recognises both α and β linked galactose indicates either a broad specificity galactose binding lectin or two or more lectins with restricted specificity. As binding to these different galactose structures is not preferential under any condition tested, it is likely that a single yet to be identified broad specificity glactose binding lectin is expressed by *C. jejuni*.

Fucose is a known chemoattractant of *C. jejuni* but the binding observed in our glycan array analysis is unlikely to be related to the periplasmic receptors for chemotaxis. Fucose surface expression in humans is dependent on a range of fucosyltransferases that can be differentially expressed both throughout tissues and between individuals resulting in differential fucosylation between tissue types or differential fucosylation of the same tissue types when comparing two nonrelated individuals. As *C. jejuni* has no preference for linkage or location it is likely that either the same protein that recognises galactose is binding fucosylated structures but ignoring the presence of fucose or that *C. jejuni* has a broad specificity fucose binding lectin.

Binding to N-acetylglucosamine structures was differential between strains with three strains not recognising GlcNAc structures at all (*C. jejuni* 11168, 019 and 108). Typically among strains that did recognise GlcNAc structures the longer repeats were preferred. Only *C. jejuni* 331 (under all conditions), 81116 (under all conditions) and 351 (under environmental conditions) recognised the short repeats. Chitin a common glycoconjugate found in insects and crustaceans is comprised of repeating GlcNAc residues. It is possible that *C. jejuni* strains that recognise GlcNAc structures may use insects as vectors as described by Hald *et al.*[19], or that strains with GlcNAc recognition can better infect crustaceans to survive and propagate in fresh water ponds and streams [19,20]. Chitin recognition may therefore be important for environmental survival and spread, also offering advantages for re-infection of more preferred avian or mammalian hosts.

In line with previously reported data [3], mannose was recognised more often after environmental stress by most of the *C. jejuni* strains tested. *C. jejuni* 331 and 81116 were the only strains to recognise a wide variety of mannose structures under all growth/maintenance conditions. Several other strains, more common to the chicken isolates tested (Human isolate: *C. jejuni* 351; Chicken isolates: *C. jejuni* 108, 434 and 506), also recognised some of the branched mannose structures under all conditions tested. Branched mannose is far more common in complex N-linked glycans found on many different cell surface proteins. These branched mannose structures are typically capped by other sugars including Glc/GlcNAc, Gal/GalNAc and sialic acid implying that either these interactions are through subterminal binding proteins that can recognise capped structures or are not biologically relevant to infection/colonisation. From the binding profile of *C. jejuni* to the complex sialylated structure, 11D, it appears in all cases but *C. jejuni* 108 that subterminal recognition of mannose in complex N-linked glycans can be ruled out.

Similar to *C. jejuni* binding to mannose, sialic acid recognition was only observed following a period of environmental stress, with all the *C. jejuni* strains tested exhibiting significantly more binding to sialylated glycans when maintained under normal atmosphere and at room temperature. This indicates that an adhesion/lectin able to bind sialylated glycans is regulated by the exposure of *C. jejuni* to environmental stress. As yet, no such protein has been elucidated in *C. jejuni*. Sialic acid is a common glycan present on multiple cell types and is typically the terminal sugar presented. In the intestines MUC1 is the most heavily sialylated protein present, however, MUC1 acts as a decoy receptor for bacteria and other viral and microbial infecting agents [10]. When MUC1 is bound by pathogens it is released from the cell surface and allows the pathogen to be excreted into the environment through the lumen [10]. A number of pathogens, including *C. jejuni*, are more infectious, have a lower infectious dose or get into deeper tissues faster when administered to MUC1^−/−^ mice [10].

Of the few sialylated structures that were bound more broadly by *C. jejuni,* 10A (*C. jejuni* strains 351, 375, 520, 331, 434, 506), 10B (*C. jejuni* strains 351, 375, 520, 331, 434, 506) and 10D (all strains tested), are all fucosylated, indicating that the binding to these glycans may be more due to fucose than to sialic acid. *C. jejuni* 81116, once again, recognised a wider variety of sialic acid containing structures than the other *C. jejuni* strains tested, binding to α2-3 linked sialylactosamine structures. *C. jejuni* 81116 has a vastly different cell surface glycosylation profile than other *C. jejuni* producing larger non-sialylated LPS like molecule rather than the traditional LOS seen for other *C. jejuni*[21]. It may be interesting to speculate that surface glycosylation can play a role in the inhibition of the binding of *C. jejuni* to sialylated glycans, particularly through charge-charge repulsion. Sialic acid is a negatively charged sugar and *C. jejuni* strains such as 11168 are known to have surface glycosylation that contains sialic acid [22,23]. Of the strains that bound to sialyllewis structures (10A and B), we have recently shown that, *C. jejuni* 351, 375 and 331, do not have surface sialylation [24], indicating these strains may be able to recognise the underlying fucose. We are yet to confirm the sialylation levels of *C. jejuni* strains 434 and 506. *C. jejuni* 520 seems to be a special case as the LOS it produces appears to be very heterogenous [24]. We have shown using lectin array and surface plasmon resonance that a proportion of the LOS produced by this strain is completely non-sialylated at all growth conditions tested [24]. It is therefore possible that sufficient *C. jejuni* 520 was present in the assay with low or no surface sialylation allowing for recognition of the underlying branched fucose.

Glycoaminoglycan binding by *C. jejuni* on glycan arrays has not previously been reported. *C. jejuni* in general preferred larger GAG fragments, with the most consistent binding observed to full length GAGs of up to 1.6MDa. GAGs are common extracellular matrix components and are expressed in on the surface of a broad range of cells [25-27]. GAGs are also known to associate with known cell surface targets of *C. jejuni* including fibronectin [25-27]. Once more 81116 had the broadest recognition for GAG and related structures recognising all the structures present on our array.

The non-invasive *C. jejuni* strain 331 had a preference for longer, branched galactose structures and was less likely to associate with disaccharides or terminal N-Acetylgalactosamine structures. This is of interest as *C. jejuni* 331 is known to be a strong chicken coloniser, capable of out competing other *C. jejuni* strains in co-infection studies and has been proposed as a potential non-virulent bioreplacement bacteria [28,29]. It is possible that the lack of binding to disaccharide and small sugar subunits by *C. jejuni* 331 may offer a competitive advantage, allowing 331 to better colonise the intestinal crypts by ignoring smaller sugars in the lumen. Mono- and di-saccharides are common products from the activity of glycosidases in the intestinal tract of animals. This makes mono- and di-saccharides potential decoy receptors for *C. jejuni* in the chicken gut and as such, bacteria that do not bind to smaller sugars would potentially have a competitive advantage.

## Conclusions

The conclusions drawn from the initial screening of *C. jejuni* 11168 on our glycan array [3] have in the main been confirmed by the screening of additional strains. Sialic acid and mannose still appear to be the general structures recognised after environmental stress, appearing to be important for initial host pathogen interactions. Galactose and fucose structures still appear to be crucial for the persistence of infection. Little difference is seen between the isolates from clinical or chicken hosts, with the exception of carageenan and branched mannose binding, with both more likely to be recognised by chicken isolates than those isolated from humans. This study increases the understanding of *C. jejuni* glycan recognition and provides a model for the study of complex glycan recognition from a number of other yet to be screened bacterial species.

## Methods

### Bacterial strains and growth conditions

The strains used in this study can be found in Table 5. Bacteria were grown as previously described [3].

**Table 5:** Bacterial strains used in this study

**Strain**	**Invasive**	**Source**
Human	+/−	
11168	+	D. Newell
351	+	RMIT
375	+	RMIT
520	+	RMIT
81116	+	D. Newell
81-176	+	J. G. Fox
Chicken		
331	-	RMIT
8	+	RMIT
19	+	RMIT
108	+	RMIT
434	+	RMIT
506	+	RMIT

### Glycan arrays

Glycan arrays were prepared and performed as previously described by Day *et al.*[3] with slight modification to the preparation of the slides as outlined by Hartley-Tassell *et al.*[30] using the glycan library described in Arndt *et al.*[3,30,31]. See Additional file 1: Table S1 for full list and structures of glycans. The arrays were scanned by a ProScan Array scanner at 488/520 nm and the results analysed by ScanArray Express software program. Binding was defined as a value greater than 1 fold increase above mean background relative fluorescence units (RFU). The mean background was calculated from the average background of empty spots on the array plus three standard deviations. Statistical analysis of the data was performed by a Student’s t-test with a confidence level of 99.99% (p ≤ 0.0001). All arrays were performed in triplicate with a total of 12 data points for each glycan tested.

### Lectin competition adherence assays

Adherence and lectin competition assays were performed as previously described [3], however, only using *C. jejuni* grown at 37°C under micraerobic conditions. *E. coli* DH5α cells were used as a control for the lectin competition assays to ensure that reduction in adherence was not due to steric hindrance of the lectins on the cell surface inhibiting cell binding to non-glycan targets. Lectins were used at 10 μg per well. All assays were performed in triplicate.

### Free glycan inhibition assay

Adherence assays were performed as previously described [3] under conditions described above. Two exemplary strains of *C. jejuni* were used for this analysis, the human clinical isolate 11168 and the chicken isolate 331. Free glycan (H-disaccharide, A-blood group antigen and α1-2 mannobiose) were added to the media at a final concentration of 100 μM just prior to addition of the bacteria.

## Competing interests

The authors declare that they have no competing interests.

## Authors’ contributions

CJD conceived the experiments, performed many of the array and all the cell culture experiments and aided in the analysis of the data. CJD wrote a significant portion of the completed manuscript. GT helped perform array experimentation, aided with the glycan inhibition cell culture assays, helped analyse data and aided in the production of the manuscript. LEH-T helped performed array experimentation, helped analyse data including the establishment of the statistical template and aided in the production of the manuscript. JT helped performed array experimentation, helped analyse data and aided in the production of the manuscript. VK conceived the experiments, aided in the analysis of the data and was responsible for final edits of the completed manuscript. All authors read and approved the final manuscript.

## Supplementary Material

Additional file 1 Table of Glycans. Click here for file

## References

[B1] Smith DC Lord JM Roberts LM Johannes L Glycosphingolipids as toxin receptors Semin Cell Dev Biol 2004 15 4 397 408 10.1016/j.semcdb.2004.03.005 15207830

[B2] Lehmann F Tiralongo E Tiralongo J Sialic acid-specific lectins: occurrence, specificity and function Cell Mol Life Sci 2006 63 12 1331 1354 10.1007/s00018-005-5589-y 16596337 PMC7079783

[B3] Day CJ Tiralongo J Hartnell RD Logue CA Wilson JC von Itzstein M Korolik V Differential carbohydrate recognition by *Campylobacter jejuni* strain 11168: influences of temperature and growth conditions PLoS One 2009 4 3 e4927 10.1371/journal.pone.0004927 19290056 PMC2654152

[B4] Day CJ Semchenko EA Korolik V Glycoconjugates play a key role in *campylobacter jejuni* infection: interactions between host and pathogen Front Cell Infect Microbiol 2012 2 9 22919601 10.3389/fcimb.2012.00009 PMC3417407

[B5] Newburg DS Ruiz-Palacios GM Morrow AL Human milk glycans protect infants against enteric pathogens Annu Rev Nutr 2005 25 37 58 10.1146/annurev.nutr.25.050304.092553 16011458

[B6] Morrow AL Ruiz-Palacios GM Jiang X Newburg DS Human-milk glycans that inhibit pathogen binding protect breast-feeding infants against infectious diarrhea J Nutr 2005 135 5 1304 1307 15867329 10.1093/jn/135.5.1304

[B7] Yamaoka Y Roles of Helicobacter pylori BabA in gastroduodenal pathogenesis World J Gastroenterol 2008 14 27 4265 4272 10.3748/wjg.14.4265 18666312 PMC2731175

[B8] Juge N Microbial adhesins to gastrointestinal mucus Trends Microbiol 2012 20 1 30 39 10.1016/j.tim.2011.10.001 22088901

[B9] Mahdavi J Sonden B Hurtig M Olfat FO Forsberg L Roche N Angstrom J Larsson T Teneberg S Karlsson KA *Helicobacter pylori* SabA adhesin in persistent infection and chronic inflammation Science 2002 297 5581 573 578 10.1126/science.1069076 12142529 PMC2570540

[B10] McAuley JL Linden SK Png CW King RM Pennington HL Gendler SJ Florin TH Hill GR Korolik V McGuckin MA MUC1 cell surface mucin is a critical element of the mucosal barrier to infection J Clin Invest 2007 117 8 2313 2324 10.1172/JCI26705 17641781 PMC1913485

[B11] Varki A Multiple changes in sialic acid biology during human evolution Glycoconj J 2009 26 3 231 245 10.1007/s10719-008-9183-z 18777136 PMC7087641

[B12] Le Pendu J Histo-blood group antigen and human milk oligosaccharides: genetic polymorphism and risk of infectious diseases Adv Exp Med Biol 2004 554 135 143 10.1007/978-1-4757-4242-8_13 15384573

[B13] Suzuki N Laskowski M Jr Lee YC Phylogenetic expression of Galalpha1-4Gal on avian glycoproteins: glycan differentiation inscribed in the early history of modern birds Proc Natl Acad Sci USA 2004 101 24 9023 9028 10.1073/pnas.0402822101 15184685 PMC428466

[B14] Galili U Clark MR Shohet SB Buehler J Macher BA Evolutionary relationship between the natural anti-Gal antibody and the Gal alpha 1––3Gal epitope in primates Proc Natl Acad Sci USA 1987 84 5 1369 1373 10.1073/pnas.84.5.1369 2434954 PMC304431

[B15] Yang Z Bergstrom J Karlsson KA Glycoproteins with Gal alpha 4Gal are absent from human erythrocyte membranes, indicating that glycolipids are the sole carriers of blood group P activities J Biol Chem 1994 269 20 14620 14624 8182069

[B16] Sandrin MS McKenzie IF Gal alpha (1,3)Gal, the major xenoantigen(s) recognised in pigs by human natural antibodies Immunol Rev 1994 141 169 190 10.1111/j.1600-065X.1994.tb00877.x 7532618

[B17] Garratty G Blood group antigens as tumor markers, parasitic/bacterial/viral receptors, and their association with immunologically important proteins Immunol Invest 1995 24 1–2 213 232 7713584 10.3109/08820139509062774

[B18] Houliston RS Vinogradov E Dzieciatkowska M Li J St Michael F Karwaski MF Brochu D Jarrell HC Parker CT Yuki N Lipooligosaccharide of Campylobacter jejuni: similarity with multiple types of mammalian glycans beyond gangliosides J Biol Chem 2011 286 14 12361 12370 10.1074/jbc.M110.181750 21257763 PMC3069439

[B19] Hald B Skovgard H Pedersen K Bunkenborg H Influxed insects as vectors for Campylobacter jejuni and Campylobacter coli in Danish broiler houses Poult Sci 2008 87 7 1428 1434 10.3382/ps.2007-00301 18577626

[B20] Schallenberg M Bremer PJ Henkel S Launhardt A Burns CW Survival of Campylobacter jejuni in water: effect of grazing by the freshwater crustacean Daphnia carinata (Cladocera) Appl Environ Microbiol 2005 71 9 5085 5088 10.1128/AEM.71.9.5085-5088.2005 16151090 PMC1214637

[B21] Holden KM Gilbert M Coloe PJ Li J Fry BN The role of WlaRG, WlaTB and WlaTC in lipooligosaccharide synthesis by Campylobacter jejuni strain 81116 Microb Pathog 2012 52 6 344 352 10.1016/j.micpath.2012.03.004 22445818

[B22] St Michael F Szymanski CM Li J Chan KH Khieu NH Larocque S Wakarchuk WW Brisson JR Monteiro MA The structures of the lipooligosaccharide and capsule polysaccharide of Campylobacter jejuni genome sequenced strain NCTC 11168 Eur J Biochem 2002 269 21 5119 5136 10.1046/j.1432-1033.2002.03201.x 12392544

[B23] Semchenko EA Day CJ Wilson JC Grice ID Moran AP Korolik V Temperature-dependent phenotypic variation of Campylobacter jejuni lipooligosaccharides BMC Microbiol 2010 10 305 10.1186/1471-2180-10-305 21118497 PMC3009654

[B24] Semchenko EA Day CJ Moutin M Wilson JC Tiralongo J Korolik V Structural heterogeneity of terminal glycans in Campylobacter jejuni lipooligosaccharides PLoS One 2012 7 7 e40920 10.1371/journal.pone.0040920 22815868 PMC3397941

[B25] Yamada KM Kennedy DW Kimata K Pratt RM Characterization of fibronectin interactions with glycosaminoglycans and identification of active proteolytic fragments J Biol Chem 1980 255 13 6055 6063 6771264

[B26] Konkel ME Garvis SG Tipton SL Anderson DE Jr Cieplak W Jr Identification and molecular cloning of a gene encoding a fibronectin-binding protein (CadF) from Campylobacter jejuni Mol Microbiol 1997 24 5 953 963 10.1046/j.1365-2958.1997.4031771.x 9220003

[B27] Chung CY Erickson HP Glycosaminoglycans modulate fibronectin matrix assembly and are essential for matrix incorporation of tenascin-C J Cell Sci 1997 110 Pt 12 1413 1419 9217327 10.1242/jcs.110.12.1413

[B28] Calderon-Gomez LI Hartley LE McCormack A Ringoir DD Korolik V Potential use of characterised hyper-colonising strain(s) of Campylobacter jejuni to reduce circulation of environmental strains in commercial poultry Vet Microbiol 2009 134 3–4 353 361 18977611 10.1016/j.vetmic.2008.09.055

[B29] Korolik V Alderton MR Smith SC Chang J Coloe PJ Isolation and molecular analysis of colonising and non-colonising strains of Campylobacter jejuni and Campylobacter coli following experimental infection of young chickens Vet Microbiol 1998 60 2–4 239 249 9646454 10.1016/s0378-1135(98)00145-x

[B30] Hartley-Tassell LE Shewell LK Day CJ Wilson JC Sandhu R Ketley JM Korolik V Identification and characterization of the aspartate chemosensory receptor of Campylobacter jejuni Mol Microbiol 2010 75 3 710 730 20025667 10.1111/j.1365-2958.2009.07010.x

[B31] Arndt NX Tiralongo J Madge PD von Itzstein M Day CJ Differential carbohydrate binding and cell surface glycosylation of human cancer cell lines J Cell Biochem 2011 112 9 2230 2240 10.1002/jcb.23139 21480363

